# Carbon Nanotubes: Solution for the Therapeutic Delivery of siRNA?

**DOI:** 10.3390/ma5020278

**Published:** 2012-02-13

**Authors:** D. Lynn Kirkpatrick, Michelle Weiss, Anton Naumov, Geoffrey Bartholomeusz, R. Bruce Weisman, Olga Gliko

**Affiliations:** 1 Ensysce Biosciences Inc., 7000 Fannin St., Suite 2115, Houston, TX 77030, USA; E-Mails: mweiss@ensysce.com (M.W.); anaumov@ensysce.com (A.N.); ogliko@ensysce.com (O.G.); 2 MD Anderson Cancer Center, 1901 East Road, Unit 1950, Houston, TX 77054, USA; E-Mail: gbarthol@mdanderson.org; 3 Department of Chemistry, Rice University, 6100 Main Street, Houston, TX 77005, USA; E-Mail: weissman@rice.edu

**Keywords:** single-walled carbon nanotube, short interfering RNA, therapeutic delivery

## Abstract

Carbon nanotubes have many unique physical and chemical properties that are being widely explored for potential applications in biomedicine especially as transporters of drugs, proteins, DNA and RNA into cells. Specifically, single-walled carbon nanotubes (SWCNT) have been shown to deliver siRNA to tumors *in vivo*. The low toxicity, the excellent membrane penetration ability, the protection afforded against blood breakdown of the siRNA payload and the good biological activity seen *in vivo* suggests that SWCNT may become universal transfection vehicles for siRNA and other RNAs for therapeutic applications. This paper will introduce a short review of a number of therapeutic applications for carbon nanotubes and provide recent data suggesting SWCNT are an excellent option for the delivery of siRNA clinically.

## 1. Introduction 

Carbon nanotubes (CNT) have many unique physical and chemical properties that are being widely explored for potential applications in biomedicine including drug delivery systems (DDS), sensors, imaging agents and composites [1,2,3,4,5,6]. The high surface area, conductivity, high tensile strength, typical high aspect ratios as well as potentially greater adsorption abilities due to their cylindrical structure make them a novel nanomaterial for drug delivery and biomedical applications [7].

Carbon nanotubes as molecular transporters can shuttle various types of biological molecules, including drugs [1], proteins [6], DNA [8] and RNA [9] into cells. The main limitations of delivery for many therapeutic agents include poor solubility, rapid deactivation, toxicity, unfavorable pharmacokinetics (PK) and poor biodistribution. For these purposes it is important to also consider the inseparable relationship between the solubility, PK and bioavailability and toxicity of the DDS being considered. Since early 2000 carbon nanotubes have been explored as DDS and most findings suggest that they can be used safely, they protect their cargo, their PK and biodistribution can be altered to achieve localization and effective biological activity can be achieved [7,10,11]. 

Carbon nanotubes are a family of highly elongated tubular nanostructures composed of sp2-hybridized carbon atoms covalently bonded into six-membered rings. The nanotubes exist in a variety of well-defined structural forms [7]. Single walled carbon nanotubes (SWCNT) or multiwalled carbon nanotubes (MWCNT) have been each explored for biological utility. Advantages of using SWCNT are that they have a smaller diameter, are more flexible and offer additional photoluminescence that has been exploited for imaging. MWCNT present a wider surface area for payload functionalization and may allow more efficient internal encapsulation. The material shows physiochemical properties which are dependent on size, chemical composition, surface structure, solubility, shape and aggregation. These parameters can modify cellular uptake, protein binding, translocation and toxicity. The lack of any consistent fabrication of CNT with structurally and chemically controlled processes unfortunately limits the ability to compare the literature with respect to many of the studies that have reported on this DDS.

### 1.1. Therapeutic Uses of Carbon Nanotubes

Therapeutic uses of CNT have been expanded over the last decade as the properties of the CNT have become more fully appreciated. Therapeutics with limitations such as poor solubility, rapid deactivation and unfavorable PK resulting in limited biodistribution require DDS and the use of CNT to overcome these issues has been explored by numerous laboratories [1,2,3,6,8,9,12,13,14,15,16]. Therapeutic delivery of chemotherapeutic agents using SWCNT have reported reduced toxicity [3], greater biological activity [1], targeting ability [17,18] and controlled release [19]. Reports have also shown that both SWCNT and MWCNT have utility for the delivery of oligonucleotides [8,12,13,14,15]. Not only do CNT carry the oligo payload into cells, they provide a protection from degradation while in the circulation [20]. Additionally, the intrinsic fluorescence of SWCNT in the NIR region provides an opportunity for imaging when these are used as a delivery vehicle [4,5,21,22,23]. SWCNT have been developed into sensing devices for human disease including for the detection of lung cancer and kidney disease in human breath, glucose levels in diabetics and H_2_O_2_ in reactive oxygen signaling pathways [24,25,26,27]. CNT arrays have also been used to explore delivery of recombinant human bone morphogenetic protein-2 as potential implanted CNT-based materials for gene and protein based delivery [6]. Local exposure of tumors containing SWCNT to NIR light has been found to provide excellent photothermal ablation [27,28,29,30]. 

### 1.2. Toxicity

Pristine CNT lack solubility [31], have a tendency for the formation of thick and inhomogeneous bundles, have short circulation half-lives, bio-incompatibility and immunogenicity. There have been studies that have examined pristine (non-functionalized) CNT for pulmonary toxicity [32,33], intratracheal instillation [34,35], pharyngeal aspiration [36], skin exposure [37] and following subcutaneous administration [38,39]. These studies reported acute pulmonary toxicity, induction of granulomas and inflammatory reactions to the pristine CNT. The length of the tubes factors in on toxicity with shorter material more readily excreted and handled through the tissues [40,41]. Other studies using functionalized CNT reported no inflammatory response with subcutaneous administration [39] and found them to be well tolerated following i.v. administration over an extended period of time [1,40,41,42]. Hence, CNT need some type of functionalization, whether covalently or non-covalently associated with the surface for solubility and biocompatibility. This functionalization allows the preparation of soluble, well dispersed CNT samples and improves their toxicological profile. It has been found that there is an inverse correlation between toxicity and the extent of CNT functionalization. 

Complement activation of CNT has been explored *in vitro* using a number of SWCNT and double-walled carbon nanotube (DWCNT) samples [43] and with long circulating PEGylated SWCNT [44]. In their study, Salvador-Morales *et al.* [43] oxidized their samples and coated with 0.5% Triton X-100 but did not indicate the median length of the nanotubes in the sample. They also reported an inability to centrifuge or filter their samples of CNT as they were hard to resuspend which suggests that the nanotubes were not well functionalized with the coating. The conclusions were that both SWCNT and DWCNT activate the human serum complement system and the DWCNT also activate the alternative pathway but they suggest that altering the surface chemistry may diminish or eliminate these effects. In an attempt to address this, Hamad *et al.* explored more fully the complement activation with PEGylated SWCNT samples. In this study they used amino-poly(ethylene glycol)_5000_-(1,2-distearoyl-*sn*-glycer-3-phophoethanolamine (amino-PEG_5000_-DSPE) or methoxy (MEO)-PEG_5000_-DSPE to coat SWCNT. The excess PEG-conjugates were removed from the samples by filtration and the lengths were estimated by atomic force microscopy (AFM) to range from 50 to 300 nm. Human serum complement activation was observed *in vitro*, however more inconclusive results were noted *in vivo* using a rat model. Again it was speculated that inadequate surface protection in the samples may account for these findings. 

The heterogeneity of the samples being used in studies of toxicity of CNT (SWCNT, MWCNT, non-functionalized, functionalized, micron length samples, nm length samples, pristine unpurified, purified to remove bundles and contaminating metals, well dispersed or aggregated) does not allow a single conclusion to be drawn as to the toxicity of this potential DDS [40,45,46]. There are however a number of positive observations made with carefully prepared CNT solutions that have been used in biological applications [46,47,48,49]. Significantly, a number of studies found that well functionalized carbon nanotubes are stable in physiological environments and can be used with little to no toxicity *in vitro* [46] and *in vivo* in mice models [47,49]. Endpoints such as survival and clinical laboratory parameters reveal no evidence of toxicity over 4 months with the i.v. administration of well dispersed SWCNT complexes to mice despite the persistence of the functionalized SWCNT in liver and spleen for the 4 month period [49].

### 1.3. PK and Biodistribution

As with toxicity, the biodistribution and elimination of CNT depends on the surface chemistry of the SWCNT. Not only does this include the size and degree of functionalization of the CNT [41,47], it also can be altered by the length and degree of branching of functionalization entities such as polyethyleneglycol (PEG) [39,42]. Studies have shown that tissue accumulation is proportional to degree and type of functionalization and excretion route is dependent on size [40]. Chuerukuri *et al.* [50] reported a half-life (t_1/2_) of approximately 1 h following the delivery to a rabbit of SWCNT funcitionalized with Pluronic F108. Singh *et al.* [48] reported that SWCNT functionalized with dietheylentriaminepentaacetic (DTPA) and labeled with indium (^111^In) were not retained in any of the reticuloendothelial system (RES) organs and were rapidly cleared for systemic blood circulation by renal excretion. In this study the product was measured to have a 3 h t_1/2_. Lui *et* *al.* [42] examined the length and branching of PEG phospholipid analogues used for functionalization of SWCNT samples that were 100 ± 50 nm and determined circulation times were significantly altered from ~1 h with SWCNT functionalized with linear (*l*-)2K PEG to 15 h with branched (*br*-)7K PEG. The *br*-7K PEG functionalized SWCNT demonstrated relatively low uptake in the RES and near complete clearance from the main organs in approximately 2 months. The distribution of the various samples showed similar organ distribution but the *br*-7K PEG/SWCNT showed 50% less liver accumulation on day 1 than the *l*-2K PEG/SWCNT sample. Besides liver and spleen accumulation on day 1, there was found slightly more than 1% ID/g tissue in bone, kidney and intestine. There was an appreciable amount of SWCNT in feces at 8 h post injection (pi) and a small amount in bladder and kidney at 24 h pi providing evidence for biliary excretion as well as renal excretion to a lesser extent. Necropsy, histology and blood chemistry performed at 3 mo pi indicated no toxicity and there was no loss of body weight in any of the mice. They concluded that SWCNT preparations with a blood circulation half-life of 12–13 h show relatively low RES and skin accumulation together with high tumor uptake, ideal for potential cancer treatment applications.

Ruggiero *et al.* [41] examined the PK of SWCNT (mean length of 195 ± 69 nm; range up to 500 nm; ζ-potential of −8.9 ± 3.3 mV) covalently functionalized with amino groups to which were appended two fluorescent dyes [Alexa Fluor (AF)488 and AF680] and metal-ion cheland DOTA radiolabeled with ^86^Y. Use of multimodal imaging determined that the constructs were rapidly renally cleared intact by glomerular filtration with partial tubular reabsorption and transient translocation into the proximal tubular cell nuclei. Although the constructs were 10 to 20 times the 30 to 50 kDa cut-off for glomerular filtration, it was speculated that the high aspect ratio of these large entities (100:1 to 500:1) were the reason why they were cleared similarly to small molecules.

The above studies contrast those conducted by Yang *et al.* [51] who administered pristine tubes non-functionalized and suspended only in 1% tween. Yang found that in contrast to the findings with modified/functionalized CNT, the excretion of the non-functionalized SWCNT was very limited over 28 days. Additionally, the distribution pattern was altered in that the SWCNT distributed internally mainly to lung on a per gram basis as well as to the liver and spleen. It was also found that there was CNT found in the brain therefore supporting the possibility of using this platform to deliver agents through the blood brain barrier. It is interesting to note that over the 28 day period of the study there was little excretion of the SWCNT and yet none of the animals exhibited any clinical sign of abnormality from as much as a 2 mg dose. This data again supports the necessity for delivery of functionalized CNT to mask the hydrophobicity of the SWCNT and to ensure not only adequate biodistribution to desired tissues but also a reasonable clearance rate through biliary or renal routes.

### 1.4. SWCNT Delivery of siRNA

It appears from the emerging data that SWCNT, when properly solubilized and functionalized, can effectively and safely shuttle biomolecules into cells. Our laboratory has been exploring the use of carbon nanotubes as a universal DDS for siRNA and other oligonucleotides. Therapeutic applications of oligonucleotide payloads have also been explored by others using siRNA covalently linked to functionalized SWCNT, covalently linked siRNA to tethering molecules on SWCNT, and on chemically functionalized MWCNT [12,13,14,15,16]. siRNA:SWCNT+ functionalized complexes targeting CD80 and SOCS1 were shown to be effective *in vitro* and *in vivo* following systemic delivery [12]. Thiol-modified siRNA have been linked to the amine-terminated polyethylene glycol phospholipids on the sidewalls of SWCNT through cleavable disulfide bonds (SWCNT–(PL-PEG2000)-SS–siRNA). Results revealed that cellular uptake of SWCNT complex and dependent on the polyethylene glycol (PEG) chain length [13,14]. The study using MWCNT contrasted studies with SWCNT in that the diameter and length of the nanotubes were much larger (lengths of up to 2 µm and diameters of 20–30 nm) [15,16]. One study using stereotactic administration of the complexes into brains of mice and rats reported a therapeutic benefit [16].

Our SWCNT delivery of siRNA utilizes non-covalent complexation of HiPCO SWCNT in formulations that are stable and biocompatible. The SWCNT solutions are processed to provide median lengths of 300–400 nm with various components that produce circulation times ranging from minutes to hours. The complexes have been delivered systemically to animals with and without subcutaneous human tumor xenografts. The endpoints that have been observed include pharmacokinetics, toxicity, antitumor activity and target protein knockdown (KD). We have produced stable formulations with siRNA targeted to PLK1, Trx-1, HIF-1α, K-RAS, EGFR as well as a number of scrambled sequences [9]. The biological activity and protein target KD have been evaluated in a number of cell lines including MiaPaCa-2 human pancreatic carcinoma, H2122 human non-small cell lung cancer, MCF7 human breast cancer, MDA-MB-231 human breast cancer and RGM1 normal gastric mucosa. The advantages of using SWCNT to deliver siRNA include providing resistance to nuclease digestion, the stability of the complex in the presence of serum, efficient transfection reducing the amount of siRNA required for systemic delivery to produce tumor target KD and the lack of toxicity of the complex and the nanotube carrier.

In this report we present studies using optimized SWCNT solutions with consistent siRNA compositions and physical properties including size and state of dispersion. The *in vitro* data demonstrate that the siRNA/SWCNT solutions are stable in biological fluid, enter cells and release siRNA intracellularly. *In* *vivo* it has been found that the pharmacokinetic t_1/2_ of the siRNA/SWCNT can be modified through excipient content of the complexes. Most importantly, systemic delivery of the complexes *in vivo* has produced antitumor activity with tumor target knockdown with an apparent lack of toxicity. We believe based on our work and that of others that the excellent membrane penetration ability, the low toxicity, the protection afforded siRNA against breakdown in blood and the favorable biological activity seen *in vivo* will allow SWCNT to become universal transfection vehicles for siRNA and other RNAs for therapeutic applications.

## 2. Results and Discussion 

### 2.1. Processing of SWCNT Samples for Biological Evaluation

**Scheme 1:**
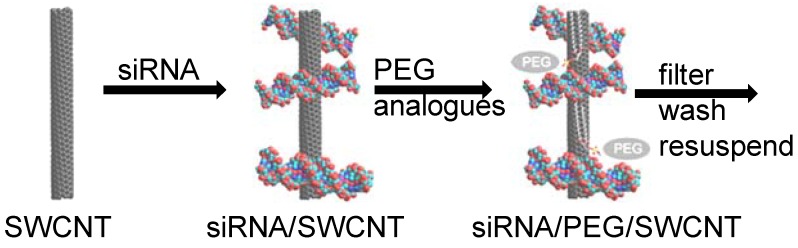
Preparation of siRNA/SWCNT or siRNA/PEG/SWCNT complexes.

The preparation of well functionalized SWCNT with siRNA has been explored using a number of variables including siRNA concentration and composition, vehicle composition, temperature, sonication time and power, Scheme 1. When different siRNA or miRNA solutions have been used as functionalizing agents there have been no differences in the preparation quality as determined by NIR and absorbance spectra measured with NS2 Nanospectralyzer (data not shown). The stability of siRNA/SWCNT samples prepared with and without filtration to remove excess siRNA from the solution was evaluated at 4 °C and room temperature. It was found that the SWCNT concentration in solution did not change over a 1 month period at either temperature. This indicated that there was still sufficient complexation by siRNA to solubilize the SWCNT. To determine whether any siRNA had dissociated from the SWCNT, the solutions were refiltered at a 1 week time point and the filtrate analyzed for siRNA using Nanodrop spectrophotometer. No siRNA was detected in solution (data not shown). This demonstrates that the solutions can be prepared and filtered to retain the uncomplexed siRNA, washed and resuspended to a desired SWCNT concentration and stored for a reasonable period of time without loss of siRNA from the SWCNT.

**Figure 1:**
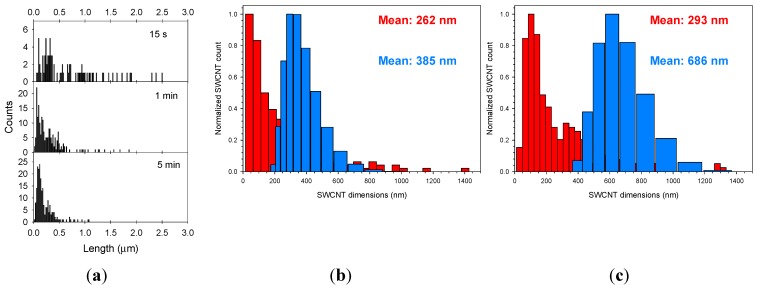
(**a**) AFM length analyses of siRNA/SWCNT complexes sonicated for 15 seconds, 1 min or 5 min; (b,c) AFM (red) and DLS (blue) length distribution and means of processed samples of (**b**) siRNA/*l*-PEG/SWCNT; (**c**) siRNA/*br*-PEG/SWCNT.

Investigation into sonication time showed a negative correlation with SWCNT length distribution (Figure 1a) with little change found following approximately 8 min of processing (not shown). In order to ensure maximum complexation of siRNA to SWCNT, sonication of siRNA and SWCNT is undertaken prior to the addition of other excipients, providing coverage of the SWCNT to approximately a 20% w/w ratio of siRNA to SWCNT. 

Analyses of the SWCNT in the samples by AFM provided an ability to determine the length distribution and coating of payload. Dynamic light scattering (DLS) was also used to determine length with the anticipation of it providing a less labor intensive method of length analyses. The samples analyzed were composed of a number of siRNA functionalizations and excipient compositions. The mean lengths as determined by DLS consistently of siRNA/*l*-PEG/SWCNT provided an over estimation of the SWCNT length of approximately 1.25 to 1.5 greater than that determined by AFM, while the overestimation of length of SWCNT processed using the *br*-PEG had the largest discrepancies of ~2.3 times greater than that determined by AFM, Figure 1b,c. This confounds the ability to use DLS alone to provide a length distribution of the prepared samples but would allow an estimate to be acquired. The zeta potential of the particles also varied depending on the composition of the coating with ζ-potential of siRNA/SWCNT particles −23.4 ± 1.0, siRNA/*l*-PEG/SWCNT −3.0 ± 1.5 and siRNA/*br*-PEG/SWCNT −6.1 ± 1.5. 

**Figure 2:**
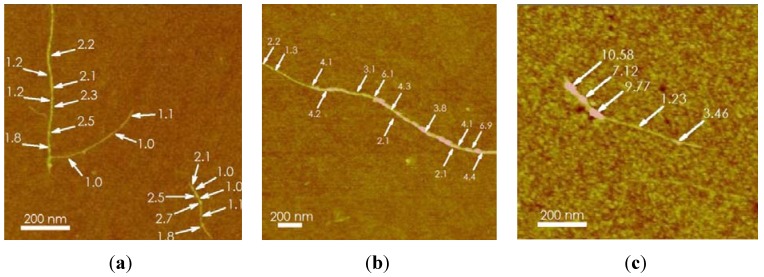
Atomic force microscopy of SWCNT complexed to (**a**) siRNA; (**b**) siRNA followed by *l*-PEG-5000; (**c**) siRNA followed by exposure to 1% bovine serum albumin (BSA). Diameters: SWCNT ~1 nm; siRNA coated ~1 to 3 nm; linear (*l*) or branched (*br*)-PEG 5000 coating ~4 to 6 nm; BSA ~7 to 10 nm.

**Figure 3:**
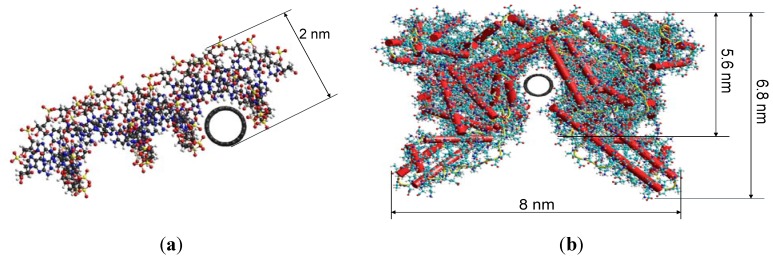
HyperChem model of (**a**) siRNA complexed to SWCNT and (**b**) human serum albumin complexed to SWCNT with diameter distances labeled.

The AFM analyses of each sample demonstrate that the SWCNT are well functionalized. Figure 2 illustrates compositions of siRNA/SWCNT, siRNA/*l*-PEG 5000/SWCNT and siRNA/SWCNT complex exposed to bovine serum albumin. These provide evidence that when siRNA/SWCNT complexes are exposed to subsequent excipients including 1% bovine serum albumin (BSA) for ~4 h, the siRNA is not displaced from the SWCNT, suggesting that *in vivo* delivery of the siRNA/SWCNT will not result in the loss of the payload in the blood. The AFM images characterize the coating efficiency, differentiating between uncoated regions and those coated by siRNA, PEG and BSA. The diameter measurements identify regions with bare SWCNT having a diameter of ~1 nm, siRNA coated >1 to 3 nm, linear or branched PEG 5000 coating ~4 to 6 nm and BSA coating areas of ~7 to 10 nm. The diameters of the siRNA coated regions and the BSA regions correspond to those of computer models of the SWCNT complexes, Figure 3. 

### 2.2. Transfection of siRNA/SWCNT Complexes

**Figure 4:**
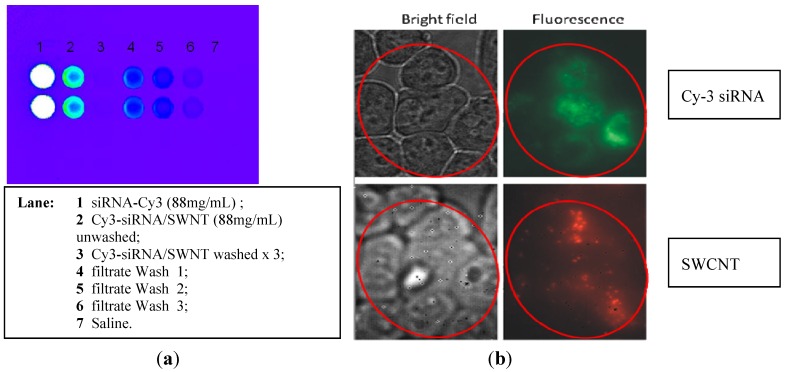
(**a**) Cy-3-siRNA complexed to SWCNT is not fluorescent (lane 3); (**b**) H2122 NSCLC cells in culture exposed to Cy-3-siRNA/SWCNT 1 µg/mL for 6 h. Cells were washed and examined by bright field and fluorescent microscopy. Top views illustrate left: the H2122 cells in culture (10.6 pix/µm); right: intracellular Cy-3-siRNA by visible fluorescence (10.6 pix/µm); and bottom views left: illustrate the same H2122 cells in culture (2 pix/µm); right: the intracellular SWCNT by NIR fluorescence (2 pix/µm) in the same cells. All cells in culture contained both the siRNA and SWCNT. Control cells not exposed to Cy-3 siRNA/SWCNT complex showed no fluorescence under either condition (data not shown).

**Figure 5:**
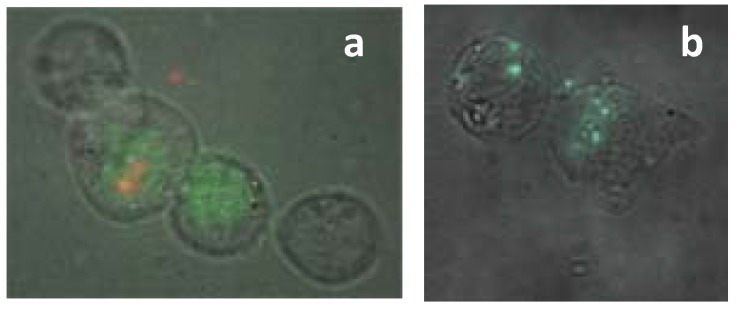
MiaCaPa-2 cells exposed to: (**a**) Cy-3-siRNA/SWCNT for 1 h. SWCNT NIR fluorescence in red and Cy-3-siRNA fluorescence in green showing siRNA distribution throughout the cells; (**b**) Cy-3-siRNA using liposomal delivery for 1 h with same amount siRNA as in (a). Green fluorescence showing the focused lipid delivery of siRNA at 1 h.

We examined the transfection of samples functionalized with siRNA alone or with different excipients. These soluble siRNA/SWCNT complexes are stable in solution and transfect siRNA *in vitro* (Figure 4 and Figure 5). It was found that when Cy-3 labeled siRNA was complexed to SWCNT the fluorescence was quenched (Figure 4a). This preparation was used to explore the release of siRNA from the SWCNT when exposed to cells *in vitro* or administered to animals *in vivo* with varying complex compositions. Cy-3-siRNA/SWCNT complexes added to either MiaPaCa-2 or H2122 NSCLC cells (data not shown) in culture, entered cells and using NIR fluorescence were observed by 1 h in cells. At 6 h post exposure to the siRNA/SWCNT complex, SWCNT were observed inside all cells (Figure 4). The same cells viewed using visible fluorescence to detect the Cy-3 labeled siRNA showed corresponding intracellular fluorescence from the siRNA, suggesting the siRNA had released from SWCNT following its transfection into the cell. It is reported that SWCNT complexes readily penetrate cell membranes by a process described as nanospearing, as tiny needles passing through the cell membrane [52], in addition to the typical endocytosis [53]. Using an equivalent amount of siRNA with liposomal delivery to compare to the SWCNT delivery revealed that at 1 h the liposomal Cy-3-siRNA remained concentrated in focused areas of the cytoplasm in contrast to SWCNT delivery which appeared to allow rapid intracellular dispersion of the siRNA (Figure 5).

### 2.3. Stability of siRNA/SWCNT Complexes 

**Figure 6:**
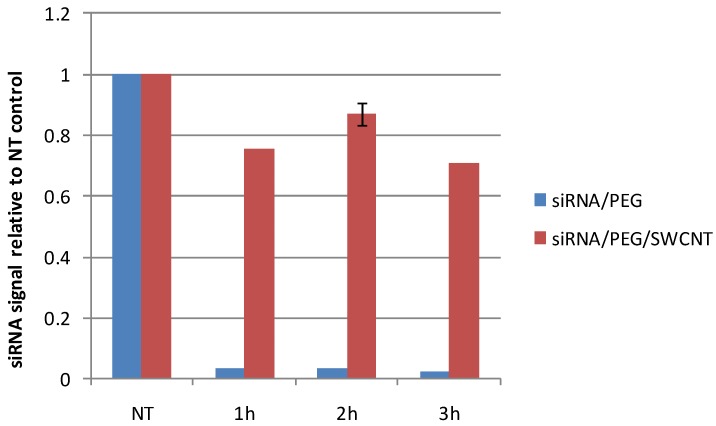
siRNA remaining after exposure to ribonuclease (10 units RNAseONE^TM^ Ribonuclease) at 37 °C for 1, 2 and 3 h in solutions of free siRNA in 64 µM PEG or siRNA complexed to SWCNT with 64 µM PEG. No treatment (NT) controls were incubated at 37 °C for 3 h. Resulting solutions were separated on agarose gels and the siRNA quantified using CareStream *In Vivo* MS FX PRO imager.

The advantages of using SWCNT to deliver siRNA include resistance to nuclease digestion, sequence-independent binding, the stability of the complex in the presence of serum, efficient transfection reducing amount of siRNA required for systemic delivery to produce tumor target knockdown (KD). We have examined the siRNA remaining from solutions of siRNA in aqueous PEG versus the same solution complexed with SWCNT when exposed to ribonuclease at 37 °C for times up to 3 h. Figure 6 shows that within 1 h the free siRNA was totally digested with less than 0.03% remaining at 1 h while approximately 80% of siRNA complexed to SWCNT remained in solution at 1 and 2 h and 70% after 3 h under same conditions.

### 2.4. Biological Activity

**Figure 7:**
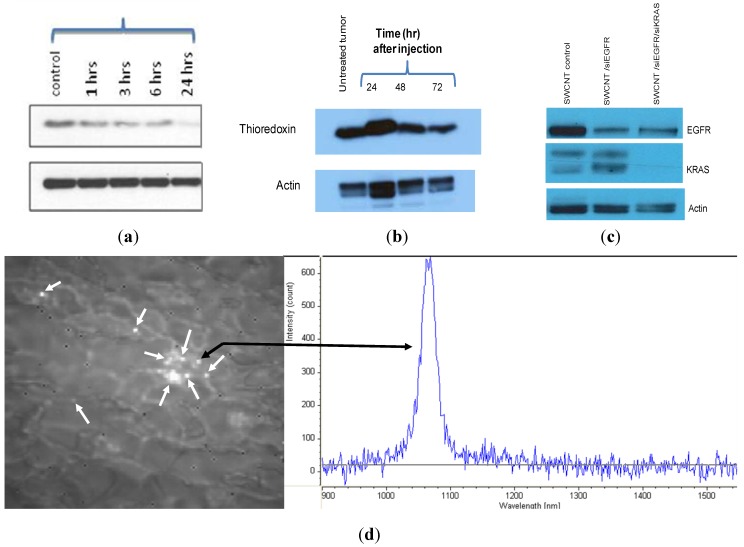
siRNA/SWCNT complexes produce time dependent target knockdown *in* *vitro* and *in vivo*. (**a**) MiaPaCa-2 cells *in vitro* with 10% FCS were exposed to siTrx(13nM)/SWCNT at times shown with Western blotting performed at 72 h. Control well: siTrx without SWCNT; (**b**) siTrx/PEG13µM/SWCNT (39 µg SWCNT, ~0.8 mg/kg siRNA) was injected into tail vein of mice bearing large MiaPaCa-2 tumors. Mice were sacrificed at 24, 48 and 72 h, tumors excised and Western blotted; (**c**) MiaPaCa-2 tumors from mice treated weekly for 4 weeks with 35 µg SWCNT carrying single siEGFR or dual payload siEGFR+siKRAS (0.8 mg/kg siRNA) (Figure 8(A)), excised and Western blotted 96 h following 4th treatment; (**d**) NIR microscopy of SWCNT in tumor slice at 24 h. Arrows identify SWCNT in tumor with corresponding characteristic NIR spectra used to distinguish SWCNT from biological fluorescing features.

**Figure 8:**
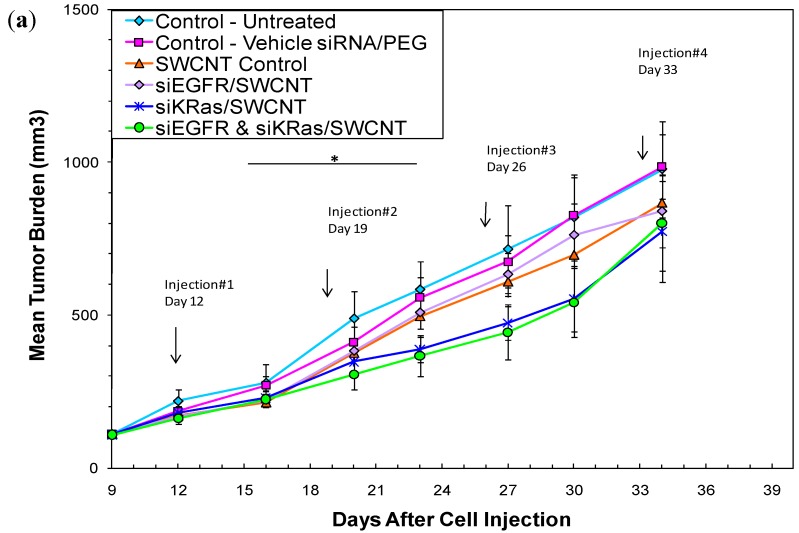

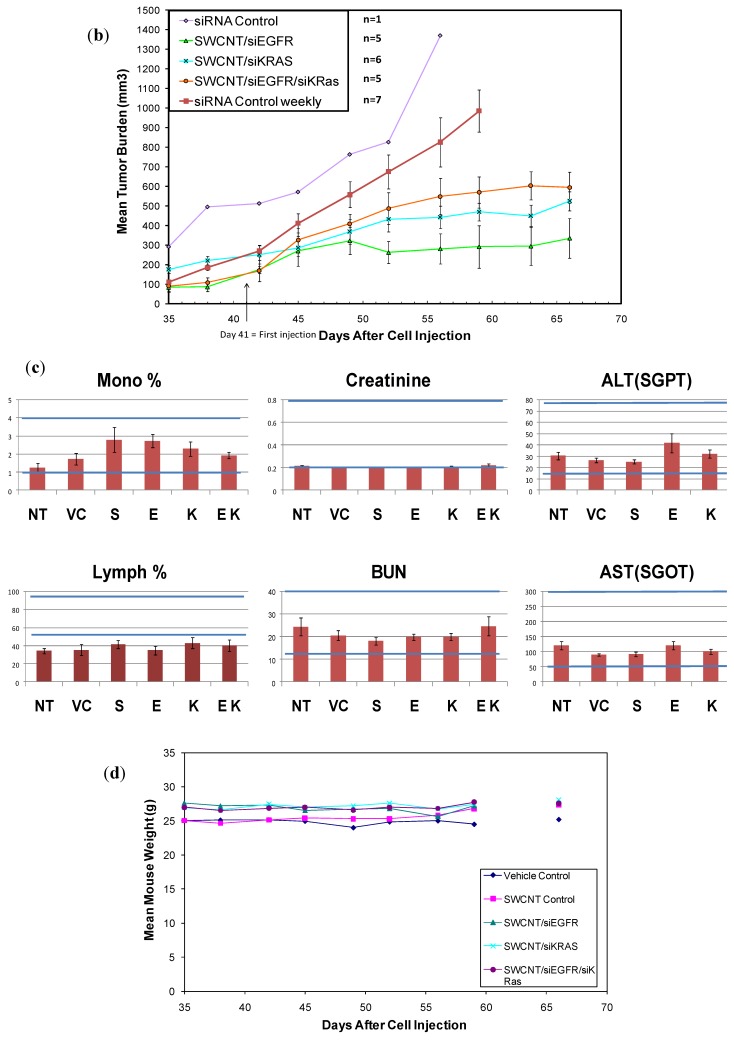
*In vivo* antitumor activity is robust with biweekly administration. Mice (8 or n) bearing MiaPaCa-2 human pancreatic tumors were treated with 35 µg siRNA/PEG 8 µM/SWCNT complexes delivering ~0.8 mg/kg siRNAs targeting EGFR, KRAS or both via tail vein injection. (**a**) Weekly for 4 weeks: *Days 15 to 23, significant difference in mean tumor growth rate siKRAS/SWCNT *vs* vehicle plus siRNA Control, *P* = 0.05; (**b**) Biweekly for 4 weeks produced a significant difference for siKRAS-, siEGFR- and the dual siEGFR/siKRAS/SWCNT treated groups from vehicle siRNA control. The siRNA control (weekly) is shown with the biweekly data since only one animal was evaluable for the siRNA control (biweekly); (**c**) Hematology and blood chemistry showed no difference when performed 24 h after final injection following weekly administration for 4 weeks. Vehicle control was siRNA and PEG 8 µM in 0.9% saline. (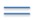 normal mouse range). NT = non-treatment control; S = SWCNT control; E = siEGFR/SWCNT; K = siKRAS/SWCNT; E K = siEGFR/siKRAS/SWCNT. Subset of data is shown; (**d**) No weight loss was observed in animals treated weekly or biweekly (not shown).

These soluble siRNA/SWCNT complexes form stable solutions that transfect siRNA *in vitro* (Figure 4, and Figure 7a) and *in vivo* (Figure 7b,c and Figure 8) and show time dependent target protein KD. MiaPaCa-2 cells in culture were exposed to siTrx/SWCNT for various times up to 24 h when media was replaced by fresh and cells were Western blotted at 72 h. Figure 7a demonstrates that biological activity (target KD) began by 1 h and increased with further exposure to the 24 h time point. Target KD was also measured *in vivo* (Figure 7b) when mice with large MiaPaCa-2 subcutaneous tumors in flank were treated by i.v. administration siTrx/PEG/SWCNT, sacrificed at 24, 48 and 72 h and tumor excised and Western blotted. Target protein, Trx KD was observed at 48 h increasing at 72 h. In a separate study, mice with MiaPaCa-2 tumors were treated with siEGFR/SWCNT or the dual payload siEGFR/siKRAS/SWCNT (Figure 7c). Tumors were excised at 96 h after i.v. administration and Western blotted for EGFR and KRAS. The target proteins were reduced in each instance where EGFR had KD in tumors of mice treated with the siEGFR/SWCNT complex and both EGFR and KRAS target protein were decreased in mice treated with the dual payload siEGFR/siKRAS/SWCNT. In addition to measuring target KD in tumors, SWCNT presence was visualized in tumor tissue slices (Figure 7d) using NIR fluorescence microscopy. The tumor content of SWCNT depend significantly on the pharmacokinetics of the SWCNT preparation, with tumors of animals treated with preparations having t_1/2_ of 10 min having 1–4 SWCNT per 150 × 125 µm while that with t_1/2_ of 25 h having an average of 14 SWCNT per 150 × 125 µm area at 24 h increasing to an average of 62 SWCNT per 150 × 125 µm area over a 96 h period. 

Additionally the antitumor activity of the siRNA/SWCNT complexes has been demonstrated (Figure 8a,b) with no apparent toxicity from SWCNT in animal models bearing human tumor xenografts (Figure 8c,d). Groups of mice received siEGFR/SWCNT, siKRAS/SWCNT or the dual payload siEGFR/siKRAS/SWCNT by i.v. administration weekly or biweekly for 4 weeks. There was a trend for reduced tumor growth rate when mice were treated weekly, but there was significant growth delay observed when they were treated with the same preparations on a biweekly schedule. Hematology and blood chemistry revealed no toxicity 24 h following the last i.v. injection on week 4 (Figure 8c) or at any time up to 96 h pi (data not shown). No weight loss was observed in any of the treatment groups whether treated weekly or biweekly (not shown).

### 2.5. Manipulating the Pharmacokinetic Properties of SWCNT

**Figure 9:**
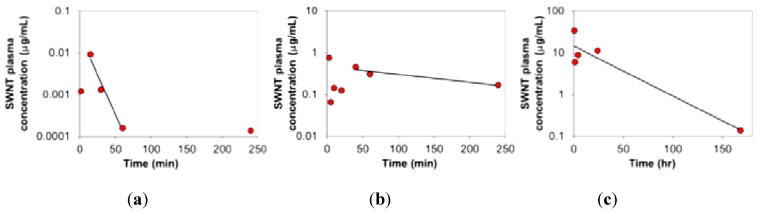
SWCNT complexed with siRNA and with excipients to modify the circulation time. (**a**) siRNA/*l*-PEG (400 µM)/SWCNT, t_1/2_ = 8–10 min; (**b**) siRNA/*l*-PEG (1600 µM)/SWCNT; t_1/2_ = 2.6 h; (**c**) *br-*PEG/SWCNT: t_1/2_ = 25 h.

**Figure 10:**
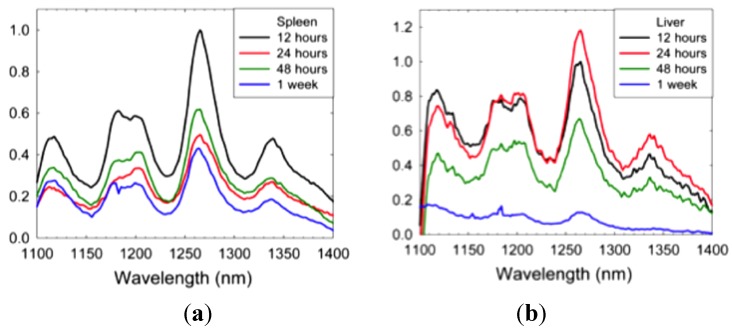
Relative SWCNT content in (**a**) spleen and (**b**) liver at 12, 24, 48 h and 1 week after mice received single bolus of 100 µg SWCNT solubilized in 3% pluronic displayed by fluorescence spectra.

Tumor localization for systemically delivered nanoparticles of siRNA relies mainly on the enhanced permeability and retention (EPR) effect and extravasation through leaky blood vessels in the tumor. However, most uptake of naked nanoparticles occurs by the RES of the liver and spleen, a process that is very rapid [54]. Attempts have been made to coat the nanoparticles with polyethyleneglycol or other materials to hide the particles from the RES (“stealth” nanoparticles), but with limited degrees of success. Attempts have also been made to prolong blood circulation time of the complexes as a more prolonged circulation of the nanoparticle is likely to favor accumulation in tumor by increasing the total number of passes made by the particle through the tumor vasculature [55]. We show that using PEG-lipid solutions in the formulation of the SWCNT complex can dramatically increase the SWCNT half-life in the plasma from minutes to 25 h (Figure 9) and increase tumor uptake. Importantly, others have shown that prolonging the plasma t_1/2_ of the SWCNT nanoparticles can increase tumor versus liver and spleen uptake [56]. Finally, our data with administration of solubilized SWCNT or siRNA/SWCNT complex confirm accumulation in tumor, with steady clearance from tissues over time (Figure 10).

The circulation time of siRNA/SWCNT complexes were measured and compared to samples with PEG added in the complexing procedure. Mice administered siRNA/SWCNT alone were administered 37 µg SWCNT. Mice administered siRNA/PEG/SWCNT complexes received 100 µg SWCNT. Figure 9 illustrates the plasma concentrations for siRNA/SWCNT complexes with *l*-PEG 400 and 1600 µM and with *br*-PEG(1 mg/mL)/SWCNT only in complexing mixture. With the injection of 100 µg siRNA/SWCNT the SWCNT were detected in the circulation up to one h pi but t_1/2_ was minutes, while with the addition of *l*-PEG to the complex the t_1/2_ could be increased based on the concentration of *l*-PEG used. *l*-PEG (400 µM) increased circulation times up to 8–10 min while *l*-PEG (1600 µM) increased the t_1/2_ to ~2.5 h. Utilizing the same size of *br*-PEG complexed directly to SWCNT with no siRNA increased the t_1/2_ to ~25 h. We are continuing to explore the compositions to provide a complex with optimal t_1/2_ and biological activity.

Although t_1/2_ of siRNA/SWCNT or siRNA/*l*-PEG/SWCNT are relatively short, tissue levels can be measured especially in liver and spleen. With tumored mice there has been SWCNT also identified in the tumor and target KD has been determined (Figure 7). Tissue elimination has been measured and was found to occur over time. Solutions of SWCNT with no payload were used for this study using 3% pluronic to solubilize. The t_1/2_ of the solution was determined to be 8.2 h. Figure 10 illustrates the fluorescent spectra of SWCNT from spleen and liver following a single 100 µg i.v. dose over a period of 1 week, demonstrating that the SWCNT are steadily eliminated from tissue. 

## 3. Experimental Section 

### 3.1. Preparation of SWCNT Solutions and SWCNT Complexes with siRNA

SWCNT (HiPco, Rice University; Lot HPR 188.4) were suspended by ultrasonic agitation in solution of 3% Pluronic^®^ F-127: Sigma Aldrich (Saint Louis, MO) to form control solutions of SWCNT alone, or with siRNA in 0.9% NaCl (siRNA targeting human EGFR (siEGFR): 5’-GAGGAAAUAUGUACUACGA[dT][dT]-3’; KRAS (siKRAS): 5’-GUCUCUUGGAUAUUCUCGA[dT][dT]-3’ or thioredoxin-1 (siTrx) 5’-CUUGGACGCUGCAGGUGAU[dT][dT]-3’ [Sigma Aldrich (Woodlands, TX, USA)] followed by centrifugation at 16,000× g for 10 min at 4 °C to remove metal contaminants or bundled SWCNT. This yielded suspensions of SWCNT coated with pluronic or siRNA. 

### 3.2. siRNA/PEG/SWCNT Complex Preparation

Raw HiPco SWCNT (4 mg) were added to 3.5 mL of siRNA (571 µg/mL) in 0.9% NaCl (siKRAS, Sigma), sonicated (Covaris S2) at 10 °C for 2 min in 15 s pulses. PL-PEG: PL-PEG 5000: 14:0 PEG5000 PE (1,2-dimyristoyl-sn-glycero-3-phosphoethanolamine-*N*-[methoxy (polyethylene glycol)-5000]ammonium salt (Avanti Polar Lipids, Alabaster, AL, USA) (*l*-PEG) at 8, 200 or 1600 µM in 0.9% NaCl or branched PEG 5000 (*br*-PEG) [43] 200 µM in 0.9% NaCl was added and sonicated for another 4 min in 15 s pulses for a total of 6 min. The samples were centrifuged at 16,000 g for 10 min at 4 °C, the supernatant was transferred into a clean tube and centrifuged for another 10 mins. The resulting supernatant contained suspension of siRNA/PEG/SWCNT complex in concentrations ranging from 129 to 184 µg/mL. The SWCNT concentration and degree of aggregation was determined by measuring absorbance at 632 nm and near-infrared fluorescence with NS2 NanoSpectralyzer (Applied NanoFluorescence). Excess siRNA and PEG were removed by filtration using 100-kDa filters (Vivaspin4, Sartorius) and washing three times with 0.9% NaCl and the complex was resuspended in 0.9% NaCl providing solutions to deliver up to 100 µg/250 µL. Size and length of complexes were analyzed by atomic force microscopy and dynamic light scattering. 

For analyses of SWCNT length as a function of time, solutions of siRNA and SWCNT as above were sonicated in 15 s pulses for up to 12 min. The samples were processed as described and the lengths were determined using AFM as described below.

#### 3.2.1. Atomic Force Microscopy (AFM)

SWCNT length distributions in the samples of various compositions were assessed using AFM and compared to those measured by dynamic light scattering. Samples were prepared as described above and diluted to ~2–5 µg/mL with 0.9% NaCl. They were deposited on the surface of 5 × 5 mm silicon AFM substrates via spin-coating. The excess of surfactant (siRNA and PEG) on the substrate was washed off by an additional spin-coating of a drop of deionized water (DI) water. The optimal surface coverage for SWCNT length and height profile measurements was achieved at 20–50 SWCNT per 5 × 5 μm region. AFM imaging was performed using a Veeco Multimode IIIA AFM with TESPA AFM cantilever tips (Bruker Scientific). Regions of 5 × 5 μm were imaged for SWCNT length distribution measurements and 1 × 1 µm regions for height profile measurements. The lengths of more than 200 SWCNT were measured for each determination using Veeco NanoScope software length measuring tool.

#### 3.2.2. Dynamic Light Scattering (DLS) and Zeta Potential 

Samples were prepared as described above, diluted to 2, 3 and 5 µg/mL with 0.9% NaCl and transferred into plastic optical cuvettes. DLS and Zeta potential measurements were performed using Malvern Zen Zetasizer Nano. Hydrodynamic dimensions of SWCNT in suspension were determined by automatic software analysis of dynamic light scattering autocorrelation curves from 32 consecutive measurements. Up to five such measurement sets were averaged and the distribution of hydrodynamic dimensions was created for each sample. Zeta potentials were measured in Malvern Instruments Folded Capillary Cells with embedded electrodes. Each measurement consisted of up to 100 runs from which the average Zeta potential value was calculated. Suspension of polystyrene nanospheres (Malvern Instruments batch 261117) with specified zeta potential of −68 ± 6.8 mV were routinely used as a standard for every set of measurements.

### 3.3. *In Vitro* Stability of siRNA/SWCNT Complexes

The concentration of each siRNA standard was determined by measuring UV absorbance at 260 nm and 280 nm using NanoDrop^TM^ 1000 spectrophotometer (Thermo Fisher Scientific). Stability of siRNA (571 µg/mL) in a solution of 64 µM PEG in 0.9% NaCl with or without complexation to SWCNT was evaluated in the presence of 10 units ribonuclease (RNAse ONE^TM^Ribonuclease, Promega, Madison, WI, USA) for 1, 2 and 3 h. After adding ribonuclease, the samples were pulse vortexed and pulse centrifuged to ensure adequate mixing, incubated at 37 °C for up to three hours. Samples and standards (18 µL of 0 to 500 µg/mL siRNA) were mixed with 4 µL 6X DNA loading dye (Fermentas R0611) and loaded on a 3% agarose gel (Sigma Aldrich, St. Louis, MO, USA). Electrophoresis was performed at 100 V for 20 min. The intensity of fluorescent signals from GelRed^TM^ (ethidium bromide substitute, Phenix Research, Chandler, NC, USA) was quantified using a Carestream Health, Inc., Rochester, NY, USA. *In Vivo* MS FX PRO imaging system at 530/600 nm excitation/emission wavelengths.

### 3.4. *In Vitro* Cellular Uptake Studies

H2122 human non-small cell lung cancer (NSCLC) cells (ATCC, US) in culture were exposed to 1 µg/mL Cy-3-siTrx/SWCNT complex for 1 to 6 h. Cells were washed and examined by bright field and fluorescence microscopy. NIR fluorescence was used to detect intracellular SWCNT and visible fluorescence to detect Cy-3-siRNA. NIR fluorescence microscopy was performed using a custom-built apparatus containing diode laser excitation sources emitting at 658 and 785 nm [57]. Cy-3 was excited with metal halide lamp and fluorescence was collected using a visible CCD camera (CoolSnap, Princeton Instruments) coupled to the microscope.

MiaCaPa-2 human pancreatic carcinoma cells were exposed to solutions of 0.2 µg/mL siRNA complexed to SWCNT or encapsulated in liposome (DharmaFECT2, Dharmacon Lafayette, CO, USA) and examined for SWCNT content via NIR fluorescence microscopy or Cy-3-siTrx via visible fluorescence as above.

### 3.5. *In Vivo* Analyses of SWCNT and siRNA/SWCNT Complexes

#### 3.5.1. Toxicity Evaluation of SWCNT with no siRNA Payload

To solubilize SWCNT without siRNA an aqueous solution of pluronic F-127 was used to complex the nanotubes. Eight-week-old C57BL6 female mice (Harlan Laboratories, US) (six mice per group) were administered sufficient volume 3% pluronic/SWCNT solution to deliver 50 and 100 µg SWCNT or the equivalent volume of aqueous 3% pluronic (vehicle) via tail vein i.v. injections. Mice were sacrificed by CO_2_ euthanasia at 24 h and 1 week post injection. Blood was obtained by cardiac puncture, plasma prepared and stored frozen at −80 °C. Blood chemistries and hematology were analyzed for each group by MD Anderson Cancer Center, Department of Veterinary Medicine & Surgery, Section of Laboratory Medicine. Tissues were harvested, flash frozen and stored at −80 °C until processed to determine SWCNT concentration.

#### 3.5.2. Pharmacokinetic Analyses of siRNA/PEG/SWCNT Solutions

siRNA/SWCNT solutions with or without PEG were administered to mice i.v. (100 µg SWCNT) into the tail vein. Mice were sacrificed by CO_2_ and blood was drawn by cardiac puncture at 3, 5, 10, 20, 60 min, 1, 4, and 24 h following injection. Blood samples were centrifuged at 1500 g for 15 min to separate optically transparent plasma from strongly absorbing red blood cells. Plasma (50 or 100 µL) was diluted in 300 µL of 2% sodium deoxycholate and sonicated for 1 s. Fluorescence signals were acquired at 637 nm laser excitation. A standard curve was generated using plasma samples, diluted with a series of 300 µL of sodium deoxycholate-SWCNT suspension of a known concentration. The fluorescence spectra in PK samples comprised independent and distinct overlapping spectra of plasma autofluorescence and SWCNT fluorescence that were fit with linear combination of plasma background and SWCNT emission profile, measured independently. This methodology allows accurate measurements of SWCNT concentration of SWCNT with optical density as low as 10^−5^. 

#### 3.5.3. Biodistribution and Elimination 

siTrx/*l*-PEG/SWCNT, *l-*PEG/SWCNT or *br*-PEG/SWCNT solutions in 0.9% NaCl were prepared to deliver 33 µg SWCNT to eight-week-old Nu/Nu female mice via tail vein i.v. injections bearing MiaPaCa-2 tumors. Mice were sacrificed by CO_2 _euthanasia at 24, 48, 72 and 96 h pi. Tissue and tumors were excised, half homogenized for Western blotting and half formalin-fixed, paraffin-embedded and sectioned in 4 µm widths for SWCNT biodistribution analyses with NIR fluorescence microscopy. 

To study elimination, 3% pluronic/SWCNT solution to deliver 50 and 100 µg SWCNT or the equivalent volume of aqueous 3% pluronic (vehicle) were administered to eight-week-old Nu/Nu female mice via tail vein i.v. injections. Mice were sacrificed by CO_2_ euthanasia at 5 min, 1, 6 and 24 h and 1 week post injection. Tissues were collected and flash frozen for future SWCNT analyses.

### 3.6. Antitumor and Toxicity Evaluation of siEGFR/siKRAS/ SWCNT Payloads

#### 3.6.1. Preparation of siEGFR/SWCNT or siKRAS/SWCNT and siEGFR/siKRAS/SWCNT

Raw HiPco SWCNT (Lot HPR 188.4), approximately 4.0 mg, were dispersed in siRNA solutions of either siEGFR or siKRAS (571.43 µg/mL) or both (1142.86 µg/mL) and these mixtures were sonicated and prepared as described above with PL-PEG at 8 µM. The SWCNT control was prepared by bath sonicating 4 mg of raw HiPco SWCNT in 4mL of 2% pluronic (F127) for 1.5 h and then tip sonicating for 2 min in 15 s bursts. Supernatant containing SWCNT solutions was removed and transferred to a sterile 15 mL conical tube and stored refrigerated at 4 °C. A 200 µL sample of each solution was analyzed using NS2 Nanaspectralyzer to determine SWCNT concentration. For i.v. administration, all solutions were bath sonicated for 1.5 h prior to filling 1cc syringe (Kendall Monoject, 1cc 28G × ½ Syringe). Solutions containing SWCNT were prepared to deliver 35 µg SWCNT in volume not exceeding 300 µL while vehicle control containing aqueous PL-PEG (8 µM) and siRNA (42.86 µM) targeting both EGFR and KRAS was administered in 200 µL volumes.

#### 3.6.2. Animal Treatment Schedule, Blood Draws and Tissue Harvesting

Eight-week-old athymic Nude-*Foxn1^nu^* female mice (Harlan Laboratories) were inoculated subcutaneously with MiaPaCa-2 human pancreatic carcinoma cells 1 × 10^7^ in 0.1mL in the right flank. Animal weight and tumor volume was measured twice weekly and tumor volumes estimated using two diameters at right angles, measured with digital calipers; Tumor volume (mm^3^) = diameter_long_ × diameter_short_^2^ × 0.5.

When tumors reached 100 mm^3^ they were randomized into groups of 10 and administered test solutions to deliver 35 µg SWCNT per dose as per study arms below, through tail vein i.v. injections once or twice per week for a total of 4 weeks. After the last injection animals (2 per group) were sacrificed by CO_2_ euthanasia at 24, 48, 72, 96 h post injection. Blood samples were collected by cardiac puncture and tissues including tumor, liver, spleen, heart, kidneys, lungs, brain, muscle, bone were flash frozen for protein knockdown and SWCNT biodistribution analyses.

Hematology and blood chemistry analyses were conducted by MD Anderson Cancer Center, Department of Veterinary Medicine & Surgery, Section of Laboratory Medicine for each group: Control-No treatment, Vehicle control, SWCNT Control, siEGFR/SWCNT, siKRAS/SWCNT, siEGFR/siKRAS/SWCNT at 24, 48, 72, 96 post final injections for 2 animals per study arm per time point.

#### 3.6.3. Tissue Analyses

**Western blotting:** Tissues were homogenized using a Polytron PT 2100 drive unit and a 5–7.5 mm dispersing aggregate in 750–1500 µL modified RIPA buffer (150 mM NaCl, 1.0% Triton X-100, 0.5% sodium deoxycholate, 0.1% SDS, 50mM Tris (pH 8.0) containing 1% EDTA-free protease inhibitor cocktail (Thermo Scientific). After approximately 10–20 seconds of homogenization, lysates were obtained by centrifugation for 20 min at 17,800 g and 4 °C. Lysates were stored at −20 °C until analyzed. Aliquots containing 30 µg protein were separated by SDS-PAGE using 12% Criterion XT Bis-Tris polyacrylamide gel (Bio-Rad, Hercules, CA), in MES/SDS Running buffer and transferred to a PVDF membrane. Western blotting was performed with specific primary antibodies against KRAS and EGFR and Actin (Santa Cruz Biotechnology, Santa Cruz, CA, USA) Membranes were exposed to anti-mouse (KRAS), anti-rabbit (EGFR) and anti-goat (Actin) HRP-conjugated-secondary antibodies in a 5% NFDM/TBS-T solution. Antibody-antigen complexes were visualized with HRP substrate chemiluminescent detection system (Perkin Elmer, Waltham, MA, USA). 

**Spectroscopy for determination of SWCNT concentration: **Tissue (0.5 g) was placed in 1.5 mL of 4% NaDOC solution and homogenized for ~1 min using homogenizer (Polytron, Kinematica) until homogeneous. Tissue homogenate, 0.5 mL was diluted 1:2 with 4% NaDOC and sonicated (S2, Covaris) at 10 °C for 30 min using 15 s pulses in 1 mL glass tube. The resulting sample was used for SWCNT concentration analysis using NS2 NanoSpectralyzer (Applied NanoFluorescence). Fluorescence signals were acquired using 637 nm laser excitation. 

**Fluorescence microscopy of tumor tissues: **Tumor tissue slices 24, 48, 72 and 96 h post injection were analyzed using custom-built NIR fluorescence microscopy setup mentioned above. Diode lasers (758 and 659 nm) were used to excite SWCNT in tissue. Their NIR fluorescence was then collected by a 60× oil immersion objective and imaged with InGaAs 2D array camera. In order to distinguish SWCNT signal from occasional tissue fluorescence, spectra of bright fluorescing spots were recorded with InGaAs spectrograph. The ones representing a single Lorentzian were considered as individual SWCNT.

## 4. Conclusions 

In summary, our biological data has demonstrated that there is no sequence dependency for the siRNA-SWCNT complex formation, and that SWCNT protect siRNA from enzymatic degradation when complexed. There is also no apparent cell line dependency for transfection, cellular uptake *in vitro* occurs rapidly and uniformly and the siRNA appears to be released from the SWCNT intracellularly. There is concentration and time dependent KD of target proteins. Multiple siRNA payloads can be delivered in one sample preparation and the complexes can be delivered systemically to produce target KD in tumors *in vivo*. Importantly, antitumor activity has been demonstrated accompanied by target KD with single and dual siRNA payloads, and the pharmacokinetic half-life can be altered by modifying payload composition. Finally, the complexes are well tolerated even with multi-day dosing for a 4 weeks period with no apparent toxicity from SWCNT in animal models bearing human tumor xenografts. Work is continuing to optimize the complex preparation and future studies will evaluate the antitumor activity of the optimized payloads.
